# Integrating Artificial intelligence within sustainable smart analytical chemistry for analyzing the divisor impact on UV-spectrophotometric efficiency of solifenacin and mirabegron combination

**DOI:** 10.1038/s41598-026-44688-x

**Published:** 2026-05-01

**Authors:** Hayam M. Lotfy, Reem H. Obaydo, Mahmoud A. Tantawy, Aya A. Mouhamed

**Affiliations:** 1 Pharmaceutical Analytical Chemistry Department, Faculty of Pharmacy, Cairo University, El-Kasr El-Aini Street, Cairo, 11562 Egypt; 2 Department of Analytical and Food Chemistry, Faculty of Pharmacy, Ebla Private University, 22743 Idlib, Syria; 3 Department of Pharmaceutical Chemistry and Pharmacognosy, College of Pharmacy, Qassim University, Buraydah, 51452 Saudi Arabia

**Keywords:** Normalized divisor (ND), Concentration dependent divisor, Extracted divisor mirabegron, Smart Analytical chemistry, Solifenacin succinate, Sustainable Smart Analytical Chemistry, Chemistry, Environmental sciences, Mathematics and computing

## Abstract

This study examines the influence of divisor selection on the efficacy of advanced analytical spectrophotometric methods that integrate artificial intelligence (AI), green-chemistry principles, and white-analytical-chemistry (WAC) frameworks for pharmaceutical investigation. Advanced analytical chemistry, which combines environmental sustainability, analytical practice and computational cleverness, was employed to create innovative spectrophotometric techniques for the concurrent quantification of solifenacin succinate (SOF), and mirabegron (MIR), both utilized in the treatment of overactive bladder. Three divisor approaches were evaluated within complementary smart resolution strategies based on high impact amplitude manipulation method (HIAM) using normalized divisor of MIR, concentration-dependent divisor of MIR at 3.0, 8.0, and 14.0 µg/mL, as well as extracted zero order spectra of MIR obtained by absorbance resolution method (AR). Linearity for SOF was observed from 2.5 to 25.0 µg/mL using first derivative D^1^at 222 nm, while MIR exhibited linearity from 1.5 to 15.0 µg/mL at its maxima 249.0 nm. To assess the robustness and risk, cumulative validation score; CVS, was calculated, serving as instrumental sign for evaluating analytical reliability and method performance. We introduce Sustainable & Smart Analytical Chemistry (SSAC), conjoining Green Analytical Chemistry (GAC), WAC, and AI to develop analytical methods that are efficient, environmentally responsible, and consistent with the multiple Sustainable Development Goals; SDGs. The Sustainability of Analytical Methods Index (SAMI) was applied to evaluate the method’s holistic alignment with the 17 SDGs. Using the Multi-Color Assessment Tool (MA), the method’s greenness, realism, presentation, and novelty were evaluated, demonstrating its sustainability and global impact.

**Supplementary Information:**

The online version contains supplementary material available at 10.1038/s41598-026-44688-x.

## Introduction

 Spectrophotometry is widely regarded as a smart analytical technique due to its simplicity, cost-effectiveness, and environmentally friendly nature. It offers rapid measurements with minimal sample preparation and low labor requirements, making it an attractive alternative to more advanced and expensive methods^1–5^. The technique can be applied through different windows, including zero-order, derivative, ratio spectra, and ratio spectra manipulations, which provide effective solutions for resolving overlapping signals in multi-component mixtures^6^. Unlike chemometric methods that require specialized software and expertise^7,8^, spectrophotometric approaches rely on straightforward mathematical procedures, ensuring accuracy, selectivity, and reproducibility while remaining accessible^9^, sustainable, and consistent with green-& white- analytical chemistry principles^6,10^.

**Fig. 1:**
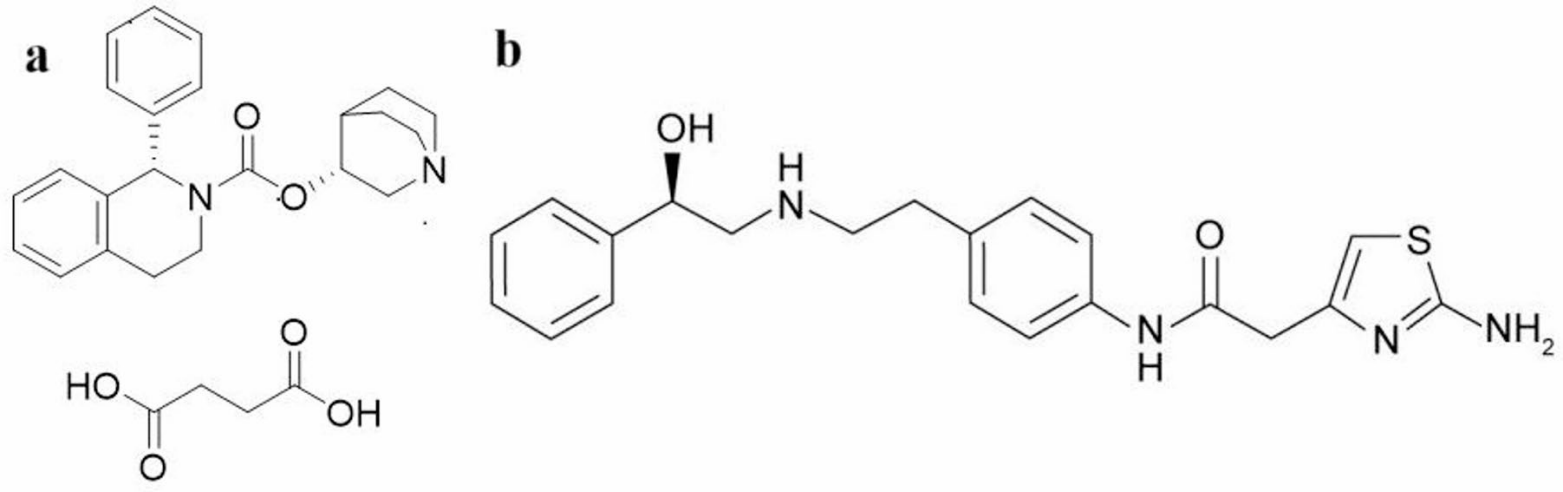
Chemical structures of studied drugs: a- Solifenacin succinate and b- Mirabegron.

Overactive bladder (OAB) is a common and uncomfortable condition that substantially impacts patients’ daily activities, often leading to social discomfort, sleep disturbances, and reduced daily functioning^11^. It affects around 16–17% of the global population, representing millions of individuals suffering from urgency, frequency, and incontinence. Among the pharmacological options, solifenacin succinate (SOF), [(3*R*)−1-azabicyclo[2.2.2]octan-3-yl](1*S*)−1-phenyl-3,4-dihydro-1*H*-isoquinoline-2-carboxylate, (Fig. 1a**)** is an antimuscarinic agent that suppresses involuntary bladder contractions, while mirabegron (MIR), 2-(2-amino-1,3-thiazol-4-yl)-*N*-[4-[2-[[(2*R*)−2-hydroxy-2-phenylethyl]amino]ethyl]phenyl]acetamide (Fig. 1b**)** is a β3-adrenoceptor agonist, relaxes the detrusor muscle to reduce urgency and incontinence episodes. MIR is clinically administered at doses of 25–50 mg once daily, while SOF is commonly prescribed at doses of 5–10 mg once daily^9^. Their fixed-dose combination has been approved to provide complementary therapeutic action and enhanced symptom control in OAB patients^11,12^.

A review of the literature revealed many techniques for concurrently assessing MIR either alone^13^ or with SOF in their combined formulations, including spectrophotometric^12,14,15^, UPLC^14^, HPLC^16^, HPTLC^17^ methods. It is worth noting that notable efforts were made in these reported spectrophotometric works regarding the selection of a suitable divisor concentration to minimize noise while maintaining optimum sensitivity^12,14,15^ Within these exerted efforts, personal error is always possible when dealing with such a large amount of data. Spectrophotometric methods represent a key solution in resource-limited laboratories, offering practical approaches for combination drug analysis while maintaining eco-friendly characteristics^18–20^.

Artificial Intelligence (AI) data analysis offers notable advantages over human analysis, including faster processing of large datasets, improved accuracy, and consistent results by reducing human error and bias. It effectively handles complex information, identifies subtle patterns, and enables scalable, high-throughput analysis. Machine learning allows AI systems to enhance their performance over time. However, AI has limitations such as lacking contextual understanding and intuition, dependence on data quality, high initial costs, and potential biases from training data, which can impact the reliability and fairness of outcomes.

As the world approaches 2030, achieving the **Sustainable Development Goals** has become a global priority, and scientific research plays a critical role in supporting this agenda^21^. Researchers are increasingly encouraged to consider how their work can contribute to **global sustainability**. In this context, C.M. Hussain et al.^22^. introduced the concept of Smart Analytical Methods (SAC), which integrates innovation in analytical chemistry with AI^23^, while incorporating GAC^24^ and WAC principles^25,26^. Users of this concept can assess alignment using algorithms such as RGB12^27,28^ and other green evaluation tools. Building on this foundation, we propose a novel framework, Sustainable & Smart Analytical Chemistry (SSAC), which explicitly links the “smart” concept to the achievement of SDGs.

To provide a comprehensive evaluation of our method’s sustainability, we employed the Sustainability of Analytical Methods Index (SAMI), an SDG-based tool that quantifies positive and negative impacts across all 17 goals through a scoring system ranging from − 2 to + 2 per goal, resulting in a normalized percentage score^29^.

Within SSAC, our study applies a UV spectrophotometric approach for the coincident analysis of two drugs by means of ethanol as a green solvent, optimized via AI to improve accuracy, efficiency, and sustainability. The method’s configuration with GAC, WAC, and innovation was evaluated using the MA tool, which provides a comprehensive whiteness score integrating greenness, practicality, analytical performance, and innovation. Finally, we demonstrate how our method contributes directly to several SDGs, thereby illustrating the potential of SSAC as a holistic, intelligent, and sustainable framework for modern analytical chemistry^30,31^.

## Challenges confronting the spectral analysis of this binary mixture

This binary mixture’s analysis, consisting of SOF and MIR in ratios of 1.0 and 5.0 respectively, faced significant challenges due to their spectral characteristics. MIR displayed a broad, intense spectrum from 200 till 400-nm, however SOF showed a weak, less-informative peak around ≈ 220-nm with minimal absorptivity outside that, close to the solvent’s cutoff. That spectral interference prevented direct measurement of MIR at its maximum and complicated the use of traditional methods that rely on constant values in extended regions, making accurate determination difficult. Moreover, standard spectrophotometric techniques struggled to distinguish SOF from MIR because of their similar spectral features.

Previous studies that used high drug concentrations further complicated analysis by causing deviations from Beer’s law, leading to unreliable results due to noise and instrument inconsistencies. Using a divisor of SOF for MIR measurement presented additional noise because of SOF’s weak absorbance, resulting in unreliable readings. The previously reported spectrophotometric methods for the determination of the SOF/MIR combination using spectrophotometric methods including absorbance resolution and ratio extraction for MIR and absorbance variation and first derivative for SOF with AI integration in the choice of the optimum wavelengths for maximum accuracy and precision, with linearity ranges of 2.5–25.0 µg/mL for SOF and 1.5–15.0 µg/mL for MIR^12^. Al-Wasidi et al.^14^ introduced approaches such as mean centering of ratio spectra, extended ratio subtraction, and the first derivative of ratio spectra. These methods did not integrate with AI and were applied to tablets, with linearity ranges of 5.0–25.0 µg/mL for both SOF and MIR. Sayed et al.^15^ presented a broader set of techniques, including dual-wavelength, induced dual-wavelength, Fourier self-deconvolution, area under the curve, ratio difference, and first derivative ratio methods. These also did not involve AI integration, but they were applied to both tablets and spiked urine samples, with linearity ranges of 70.0–1200.0 µg/mL for SOF and 50.0–450.0 µg/mL for MIR. In contrast, the current work introduced AI-integrated approaches in the analysis of SOF and MIR in combination using methods such as constant center and unified constant subtraction.

The primary objective of this research is to investigate the impact of employing different divisors (dependent concentration, independent concentration (normalized) and extracted spectrum as a divisor) on the resolution steps and overall efficiency of the analytical methods for analysis of SOF and MIR. Divisor’s choice plays serious role in balancing selectivity & sensitivity, as optimizing one can often compromise the other. Careful evaluation is necessary to ensure reliable quantification while maintaining adequate detection limits. Ultimately, selecting the appropriate divisor enhances the method’s overall robustness and applicability for pharmaceutical analysis. This evaluation is conducted through a comparative analysis of mixture results in accordance with the mandatory guidelines established by the ICH. Furthermore, the study incorporates the risk assessment via Cumulative Validation Score (CVS)^32^ which is a quantitative measure to identify the optimal divisor that ensures superior outcomes in the analysis of the selected pharmaceutical drugs. Ultimately, the findings aim to establish the most effective analytical approach that can be reliably applied to pharmaceutical formulations, thereby enhancing both accuracy and regulatory compliance. The other objective in this study is to develop Sustainable & Smart Analytical Chemistry (SSAC) by integrating Green Analytical Chemistry (GAC), white analytical chemistry (WAC), and Artificial Intelligence (AI) to create analytical methods that are efficient, environmentally responsible, and aligned with multiple Sustainable Development Goals (SDGs). This approach employs the Multi-Color Assessment Tool (MA) to evaluate and ensure the sustainability, effectiveness, and environmental impact of the proposed analytical techniques.

## Background

This resolving technique utilizes the mixture absorption spectrum’s data to accomplish separation as well as identification via spectrum factorization, employing documented signals of absorbance from zero-window (I) or ratio-window (III) within the ranges of linearity for drugs at designated wavelengths. Optimization of spectrophotometer’s parameters is essential for obtaining trustworthy findings. Disparity in data strategies utilize variations in absorbance or amplitude^33,34^.

The paternal spectra for the targeted drugs are acquired, and the X’s and Y’s concentrations are calculated using their parallel regression equations at the corresponding X’s and Y’s absorbance maxima, determined by plotting the values of absorbance within the zero-order of X’s or Y’s spectra separately at λ_max_ against the related concentrations. If the component of Y exhibits an indefinite peak, its concentration can be ascertained using a derivative technique (Δλ = 4 nm, scaling-factor = 10), with its concentration calculated through a regression equation derived from the peak’s amplitude at the derivative spectra’s maxima in relation to the corresponding concentrations.

### Absorbance resolution method coupled with Unified constant subtraction (AR-UCS)

This innovative spectrophotometric method enables precise quantification of components X and Y in binary mixtures. It involves restoring X’s parent spectrum through absorption resolution (AR) by creating a factorized-zero spectrum (FZS) based on spectrophotometric data at specific wavelengths. The process calculates the absorbance difference of Y at two wavelengths (λ1 and λ2) where this difference is effectively zero, allowing for selective analysis. A resolution tool, FS∆A, is developed by dividing X’s pure spectrum by the absorbance difference between λ1 and λ2. By measuring the mixture’s spectrum and computing the absorbance difference at these wavelengths, multiplying this by the FZS of X recovers X’s zero spectrum within the mixture. This approach effectively extracts X’s parent spectrum, enabling accurate quantification complex spectral overlaps.

The Unified Constant Subtraction method (UCS) among high-impact-amplitude-manipulation technique; HIAM^35^, applied for determination of component Y using extracted zero order spectrum of X (Using AR method). Thus, the division will give a new curve that signifies, whereas$\frac{X}{{X'}}=\,1$ then subtract from ratio spectra to get $\frac{Y}{{X'}}$ , The Y’s zero-order absorption spectrum (D^0^) (Y’s original spectrum) can be attained by multiplying the ratio spectrum $\frac{Y}{{X'}}$by X’ divisor as follows:

$$\frac{{X+Y}}{{X'}}\,\,\,=\,\,\,\frac{Y}{{X'}}\,\,\,+\,\,\,\frac{X}{{X'}}\,\,\,=\,\,\,\frac{Y}{{X'}}\,\,+\,1$$

$$=\{ \frac{Y}{{X'}}) +1\}-1\} = (\frac{Y}{{X'}})$$

$$Y =\frac{Y}{{X'}} \cdot X'$$

The AR method is limited for determine one of the component in binary mixtures at its λ_max_ where the interfering substance exhibits equal absorbance, whereas the drug of interest shows a difference. The advantage of factorized spectrum is that it is relative to the response and does not require precise preparation thus cancel random error due to sample preparation while UCS is used the other component. The unified constant extraction method offers an advantage over the constant center method by eliminating the need to calculate constant values, as it utilizes the extracted spectra of the interfering component, allowing for direct subtraction of value (one) since Y = Y’. This results in fewer manipulation steps.

### Constant-center (CC)method

CC approach is a two-step technique, comprises **(1)** applying the amplitude-difference method for the calculation of the constant, then **(2)** constant’s multiplication^35^.

Initial computation of the constant using the amplitude-difference approach relies on the presence of a combination of two medications, X and Y, with overlapping spectra. You may ascertain X’s concentration through dividing the mixture’s spectrum by a divisor (X’) of a known X concentration. The newly formed curve represented as.$\frac{X}{{X'}}+\frac{Y}{{X'}}$, Whereas$\frac{X}{{X'}}$ is a constant, whichcould be shortened as:

$$\frac{{X+Y}}{{X'}}\,\,\,=\,\,\,\frac{Y}{{X'}}\,\,\,+\,\,\,\frac{X}{{X'}}\,\,\,=\,\,\,\frac{Y}{{X'}}\,\,+\,\,cons\tan t$$

Through choosing twoλs; λ_1_& λ_2_,within attained mixture’s ratio-curve& then subtract ratios’ values at these two wavelengths λ_1_ &λ_2_ : $(\frac{Y}{{X'}})1$ and $(\frac{Y}{{X'}})2$ , cancelation of constant $\frac{X}{{X'}}$ will be achieved accompanied by any error related to instrumentation or any other effect from X’s interference, as a result cancelation of X will be attained completely, &the pure component Y will be represented by the difference;

$$\begin{gathered} P1 - P2=(\,\frac{Y}{{X'}})1\,\,+\,\,cons\tan t - \{ (\,\frac{Y}{{X'}})2\,+\,\,cons\tan t\} \hfill \\ p1 - p2=\,(\frac{Y}{{X'}})1\,\, - (\,\frac{Y}{{X'}})2\, \hfill \\ \end{gathered}$$

Compute equation representing linear correlation between ratio amplitudes’ differences of varying concentrations of pure Y at λ_1_&λ_2_, utilizing a specific concentration of X’ divisor, in relation to the corresponding ratio-amplitude at λ_1_; consequently:

$$\{ (\frac{Y}{{X'}})1 - (\frac{Y}{{X'}})2\} {\text{ }}={\text{ }}slope{\text{ }}(\frac{Y}{{X'}})1\,+\,intercept$$

Postulated amplitude Value $(\frac{Y}{{X'}})$ 1 at (λ_1_) (P_postulated_) pertaining solely to Y component in X/Y mix may be determined using the formerly derived equation, based on the differential ratio of mix’s amplitude difference at the two specified wavelengths λ_1_&λ_2_.

Constant Value (C.V.)of$\frac{X}{{X'}}$ constant may be computed through amplitude-difference technique via subtracting mixture’s ratio-spectrum amplitude$\frac{Y}{{X'}} + \frac{X}{{X'}}$ (P_recorded_) at (λ_1_), from its postulated-amplitude at (λ_1_) {ΔP _recorded−postulated_}; consequently;

$$\frac{X}{{X'}}\,\,\,=\,\,\,\{ \frac{Y}{{X'}}\,\,\,+\,\,\,\frac{X}{{X'}}\} \,1\,\, - \,\,\,\{ \frac{Y}{{X'}}\} 1\,\,$$

$$i.e.,{\text{ }}C.V.{\text{ }}={\text{ }}\left[ {{P_{recorded}}} \right]{\text{ }} - {\text{ }}{\left[ {{\text{ }}{P_{postulated}}} \right]_{}}$$

Whereas, C.V. is constant’s value $\frac{X}{{X'}}$ , P _recorded_ is mixture’s recorded peak-amplitudeat (λ_1_) and P _postulated_ is the postulated peak amplitude at (λ_1_).

X’s zero-order absorption-spectrum (D^0^) (X’s original spectrum) can be acquired through 2nd phase, known as constant-multiplication phase via multiplication of constant’s value attained$\frac{X}{{X'}}$by X’.

$$X\,\,\,=\,\,\,\frac{X}{{X'}}\,\,\cdot\,\,X'\,$$

The extracted spectrum of X is subtracted via spectrum subtraction method from grossD^0^ of binary mixture (X/Y)to acquire recovered parent Y’s D^0^.

The proposed method can be applied for determination of both components in the binary mixtures at their λ_max_ even those mixtures with a severely overlapped spectra and their spectra show no extension for one of them with certain manipulation steps.

## Experimental

### Apparatus and software

Analyses were conducted utilizing a Shimadzu; UV-1800/double-beam spectrophotometer (Kyoto-Japan), fitted with 1.00-cm cuvettes. Scans in range of 200.0 to 400.0 nm were carried out at 0.1-nm intervals.

### Samples and solvents

Pure SOF & MIR kindly obtained from Egyptian-Drug-Authority, Cairo-Egypt. The respective purities were confirmed according to the respective official methods and found to be 99.35% ± 1.67 for SOF and 100.00% ± 0.63 for MIR. MEGATAS-S 25^®^ tablets, batch no. INP25CK02, INTAS PHARMACEUTICALS LTD, India) were used as the pharmaceutical formulation, each containing 5.0 mg SOF as well as 25.0 mg MIR. Sigma-Aldrich^®^ Ethanol of HPLC-grade, served as a solvent.

### Standard solutions

SOF’s& MIR’s stock solutions; 1.0 mg/mL, were generated by precisely weighing then dissolving the respective drugs in C_2_H_5_OH in separate 50-mL flasks. Then, 100.0-µg/mL solutions for SOF &MIR were prepared by suitable dilutions. Multiple lab prepared mixes were created by precisely transferring specified quantities of the targeted substances in different ratios from their individual working solutions.

## Procedure

### Spectral properties

**Fig. 2:**
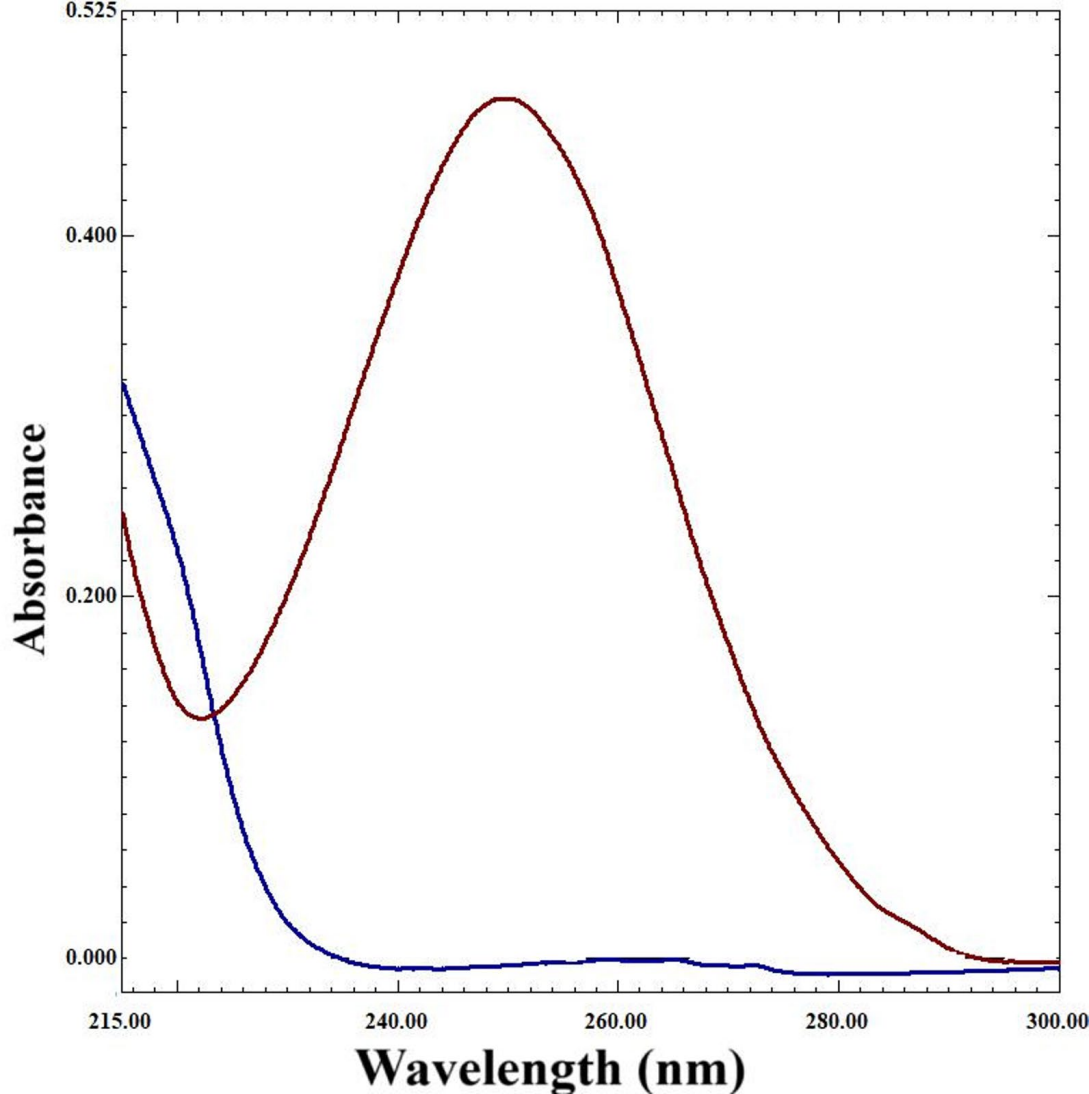
Zero order absorption spectra of 7.0 µg/mL SOF**(**^**____**^**)**and 7.0 µg/mL MIR **(**^**____**^**)**using ethanol as solvent.

SOF’s& MIR’s D^0^ were individually scanned within the λ-range of 200.0–400.0 nm, employing ethanol as the blank, and the data was stored on the computer, as depicted in Fig. 2.

### Calibration graphs construction

SOF’s& MIR’s calibration graphs were constructed by graphing(D^1^) at 222.0 nm for SOF and at 249.0 nm for MIR, alongside their equivalent concentrations2.5–25.0.0 & 1.5–15.0 µg/mL for SOF & MIR, respectively, followed by computation of regression-equations.

### Preparation and construction of divisor spectra of MIR’

#### Concentration dependent divisors

Prepare MIR standard solution of different concentrations 3.0 µg/mL(MIR’_3_), 8.0 µg/mL (MIR’_8_)and 14.0 µg/mL(MIR’_14_).

#### Normalized MIR divisor (Concentration independent divisor) (MIR’_N_)

Prepare normalized MIR using concentration of the MIR divided by its concentration.

#### Extracted MIR (MIR’_E_)

Factorized difference in absorbance spectrum for MIR(FSΔA) was produced using spectrophotometry software, which involved dividing the (D^0^) value—representing a pure MIR concentration inside its linear range—through interpoint difference in absorbance (ΔA) calculated at two λs. Specifically, for FZS method, wavelength pair chosen was A_250 nm_ and A_268 nm_.

### Wavelength selection AI-optimization & its utilization in the applied methods*

Preparation of the In-Lab application of SOF & MIR in triplicate, at a pharmaceutical-formulation ratio of 1:5, was carried out, and then tested with various divisor selections to determine ideal circumstances for the quantification of SOF and MIR. Divisors experienced Normalized divisor, Concentration-dependent divisors & Extracted divisor.

### Application to laboratory prepared mixtures

Scanning inside 215.0–300.0.0.0-nm range was conducted for each laboratory mixture. The steps of manipulation for each suggested technique were applied employing the corresponding factorized-spectrum.

#### Concentration dependent and Normalized spectrum of MIR as a divisor (CC method)

**Fig. 3:**
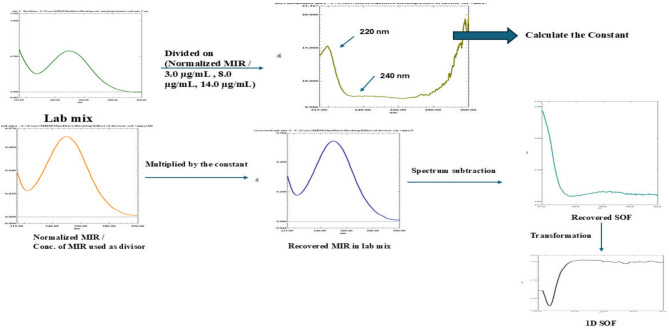
Constant Center spectrophotometric using Concentration dependent and Normalized spectra of MIR as divisors.

Division of each lab prepared mix by MIR‘(Normalized, 3.0µg/mL, 8.0µg/mL, 14.0 µg/mL) was first carried out. After that, calculation of differences in amplitude (ΔP) amid 220& 240 nm (P_220nm_-P_240nm_) Fig. 3. Calculation of postulated-amplitudes employing the agreeing regression-equations were executed with the aid of plotted ΔP (P_220nm_ - P_240 nm_) against P _maxima_ at 220 nm &values of constant were attained following subtracting mixtures’ recorded-amplitudes fromits postulated-ones at selected λ. Multiplication of MIR’s values of constant gained for each mix by MIR’ matching spectra (Normalized, 3.0, 8.0, 14.0 µg/mL), so the parent spectra of MIR in each mixture were obtained. SOF’s zero-order spectra were attained by subtraction the recovered D^0^ spectra of MIR from its parallelD^0^ of mix’s gross spectrum. SOF’s concentrations were computed from regression-equations demonstrating the amplitude of SOF at 220 nm against their concentrations.

#### Extracted spectra of MIR (AR coupled with UCS)

**Fig. 4:**
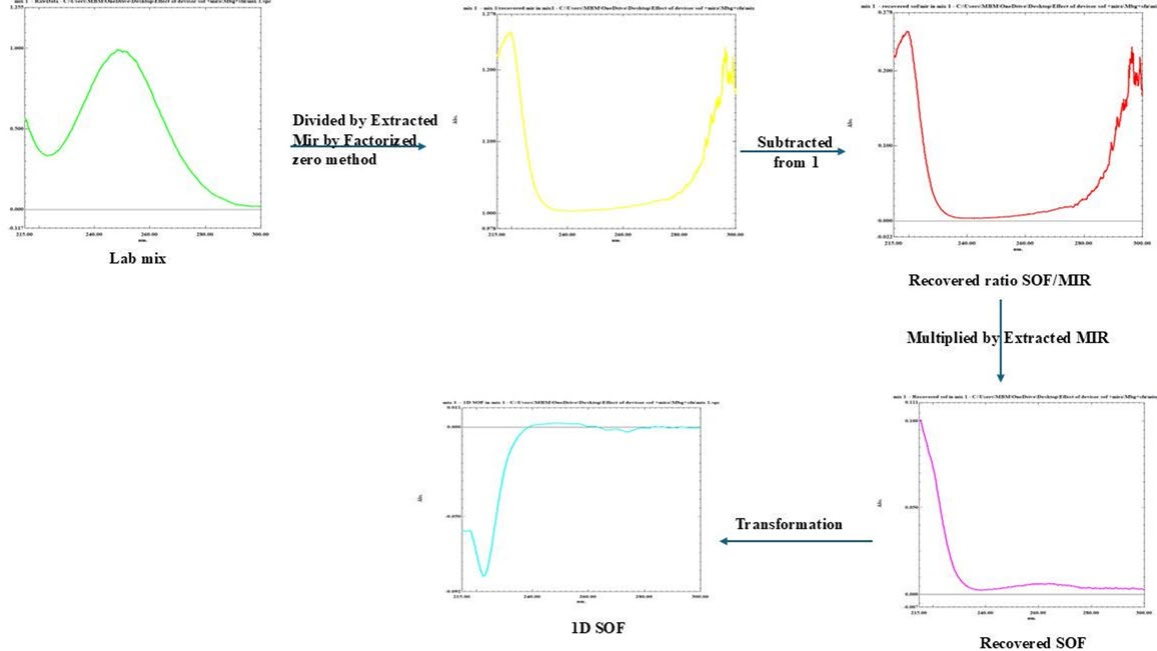
Unified Constant subtraction method using extended MIR as divisor.

Multiplication of differences in absorbance (ΔA) (A_250nm_ - A_268nm_), for each lab-prepared mix, by relevant MIR’s factorized-ΔA spectra (FS_ΔA_) set at identical λs to produce extracted D^0^spectra of MIR (Fig. 4**)**. Afterwards, the D^0^ of laboratory prepared mixture is divided by its corresponding extracted MIR spectrum. Then, subtract 1 from each ratio spectra (SOF + MIR)/MIR_E_’ of the mixture to get the ratio spectrum of SOF/MIR_E_’ then multiply by extracted MIR_E_’ to get parent spectra of SOF. The concentration of SOF is quantified through first-derivative at 222.0-nm; Δλ = 4 & scaling-factor = 10.

### Application to marketed tablets

Weighing, crushing & blending of 10 tablets were performed for uniform powder production. A portion equivalent to the weight of one tablet was carefully moved into a 50-mL flask. Addition of ethanol (≈ 25 mL) followed by 15-min sonication was carried out. After filtration, completion with the aid of ethanol was conducted. From this stock, 280 µL was taken to prepare solutions with concentrations of 2.8 &14.0 µg/mL for SOF &MIR, respectively. These solutions were examined using MIR at 14.0 µg/mL and MIR as divisors. Particular regression-equations were employed to determine drugs concentrations.

## Results and discussion

This work emphases the effect of divisors on the efficiency of spectrophotometric schemes for coincident SOF’s& MIR’s assay. The approach utilizes AI-driven signal-processing to bypass optimization phases. The project aims to enhance the efficiency and precision of SOF & MIR analyses.

### Development and optimization of proposed methods

This context investigates the impact of divisor selection on the efficiency of methods used to determine the values of SOF and MIR. The focus is on how different divisors affect the accuracy and computational cost of these methods. Specifically, the research explores whether certain divisor choices lead to more reliable, accurate, or faster outcomes when calculating SOF and MIR.

Mixtures’D^0​^ as well as ratio-spectra are classified by their degree of spectral overlap. Optimized mathematical parameters enhance selectivity, accuracy/precision as well as method’s robustness in assaying binary and/or ternary mixes with overlain spectra. It is worth noting that CVS provides an overall risk index depending on values of variability for the findings from laboratory prepared mixtures, created in twice, comprising varying quantities of pure reference conc. CVS integrates 3 components: bias (SE%) for accuracy, precision (RSD%) for repeatability, and robustness (RSD%) for wavelength shift (± 0.1 nm), with their sum representing method performance. Aggregate of these values for the examined combinations is computed regardless of the signal to obtain the CVS, with a CVS value below or equal to one (≤ 1) indicating low-risk, a value more than one & up to two (1–2) indicating intermediate-risk, and a value more than two (> 2) authorizing high-risk.

**Fig. 5:**
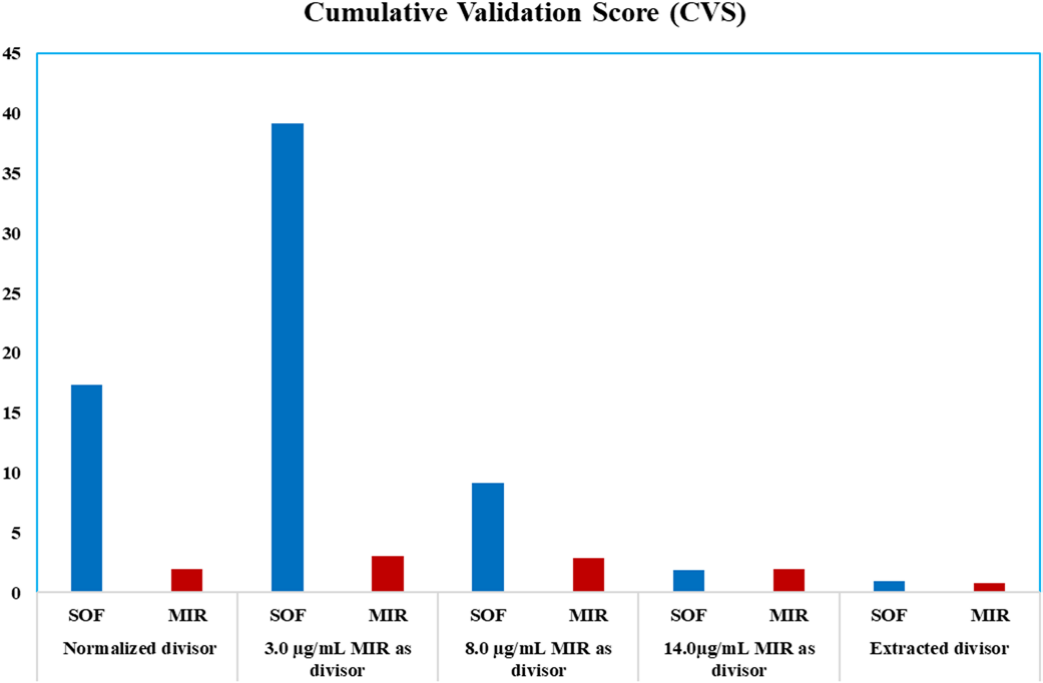
The results of Cumulative Validation Score (CVS) using constant center and unified constant subtraction spectrophotometric technique.

The CVS values were calculated for the obtained results of laboratory-prepared mixtures analysis of SOF and MIR using different divisors by the proposed spectrophotometric ratio methods Table 1; Fig. 5. For the constant center method using normalized divisor, CVS values were 17.34 for SOF and 3.50 for MIR. When using 3.0 µg/mL MIR as divisor, CVS values were 39.14 (SOF) and 3.04 (MIR)(value of CVS > 2)confirming high risk. Using 8.0 µg/mL MIR as divisor, CVS values decreased to 9.14 (SOF) and 2.92 (MIR)(value of CVS > 2) confirming high risk confirming high risk. At a higher divisor concentration of 14.0 µg/mL MIR, the CVS values further dropped to 1.88 (SOF) and 1.98 (MIR) with moderate risk (value of CVS = 1–2). Finally, with the unified constant subtraction method, the CVS values were 0.94 for SOF and 0.76 for MIR, with low risk (CVS’s value lower or equal one) demonstrating low-risk, whereas CVS’s value is higher than one, but lower than two viewing intermediate-risk, for more than two value, high-risk confirmed.

**Table 1:** Analysis of laboratory-prepared mixtures of SOF and MIR using different divisors by the proposed spectrophotometric ratio method.

**No.**	**Concentration** **(µg/mL)**	**Method**	**Constant center method**								**Unified constant subtraction **	
		**divisor**	**Normalized divisor**		**MIR 3.00 µg/mL**		**MIR 8.00 µg/mL**		**MIR 14.00 µg/mL**		**Extracted MIR**	
		**Ratio**	**SOF**	**MIR**	**SOF**	**MIR**	**SOF**	**MIR**	**SOF**	**MIR**	**SOF**	**MIR**
	**SOF: MIR**											
**1**	2.8:14.0^a^	1:5	98.73	98.02	112.56	99.35	97.58	97.82	99.88	99.20	99.73	99.74
**2**	**5.6:14.0**	2:5	89.69	101.61	124.83	100.58	101.79	98.84	98.91	99.61	99.48	99.15
**3**	**7.0:7.0**	1:1	97.10	100.53	112.30	101.97	104.47	100.12	99.56	99.66	99.86	100.15
**4**	**14.0:7.0**	2:1	97.86	99.71	106.84	101.35	101.77	99.51	99.86	99.05	99.93	100.17
**5**	**3.5:14.0**	1:4	88.20	100.58	138.89	102.12	107.56	100.38	99.42	99.75	100.11	100.30
**Laboratory Prepared mixturesMean %** ^b^± SD			94.32± 4.96	100.09± 1.34	119.09 ±12.88	101.00 ±1.14	102.63± 3.69	99.33 ±1.04	99.52±0.40	99.46± 0.31	99.82 ±0.24	99.92 ± 0.47
**Average Bias %** ^c^			5.68	0.09	19.09	1.00	2.63	0.67	0.48	0.54	0.18	0.08
**Average RSD%** ^d^			5.26	0.74	10.82	1.12	3.60	1.24	0.40	0.69	0.37	0.32
**Robustness (±0.1 nm ) RSD % ** ^e^			6.40	1.16	9.23	0.92	2.91	1.01	1.00	0.75	0.39	0.36
**Cumulative Validation Score (CVS) ** ^f^			17.34	1.99	39.14	3.04	9.14	2.92	1.88	1.98	0.94	0.76

## Data analysis and evaluation of method efficiency

In this work, the role of Artificial Intelligence (AI) is critically assessed to evaluate its effectiveness in optimizing spectrophotometric analysis. Specifically, AI (using Copilot) is employed to analyze the impact of various divisors on the efficiency of the applied method, adhering to ICH guidelines without bias. The AI system collects data such as recovery percentages, relative standard deviation (RSD%) values, and CVS (cumulative validation score) values of laboratory-prepared mixtures, which are provided in the uploaded table.

Based on this collected data, the AI evaluates the performance of each divisor in terms of accuracy and precision according to ICH regulatory. The analysis considers the recovery data to assess the method’s accuracy, while the RSD% and CVS values serve as indicators of precision and reproducibility. The AI’s role is to process these data objectively, identify trends, and recommend the most suitable divisor that ensures optimal accuracy and precision in spectrophotometric analysis.

**Figure:**
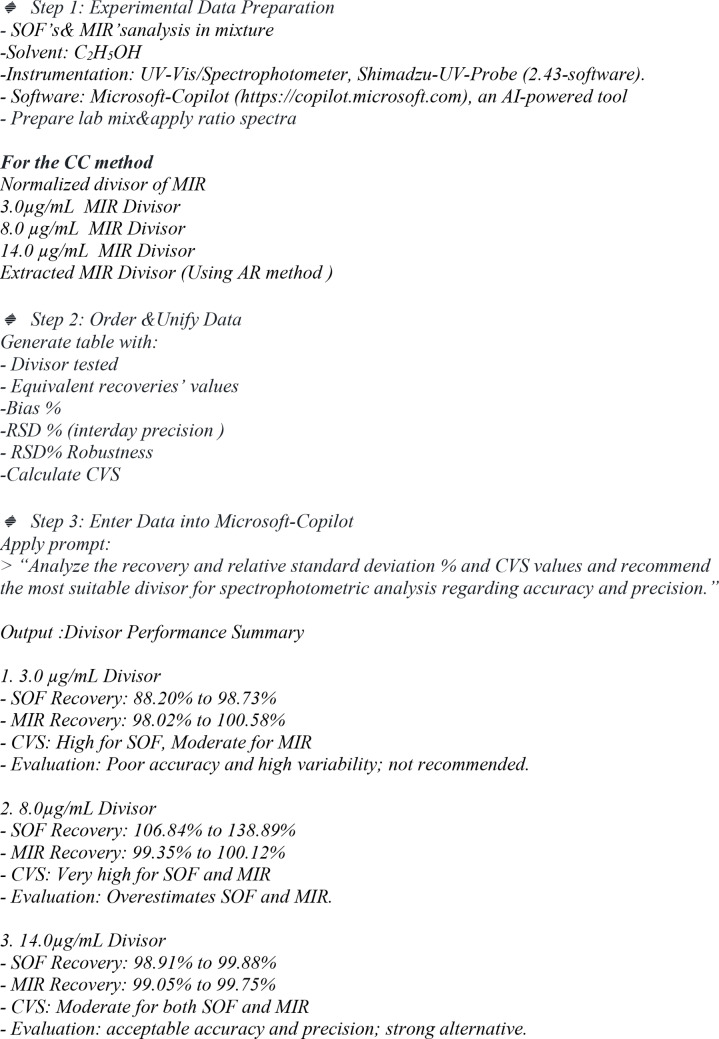

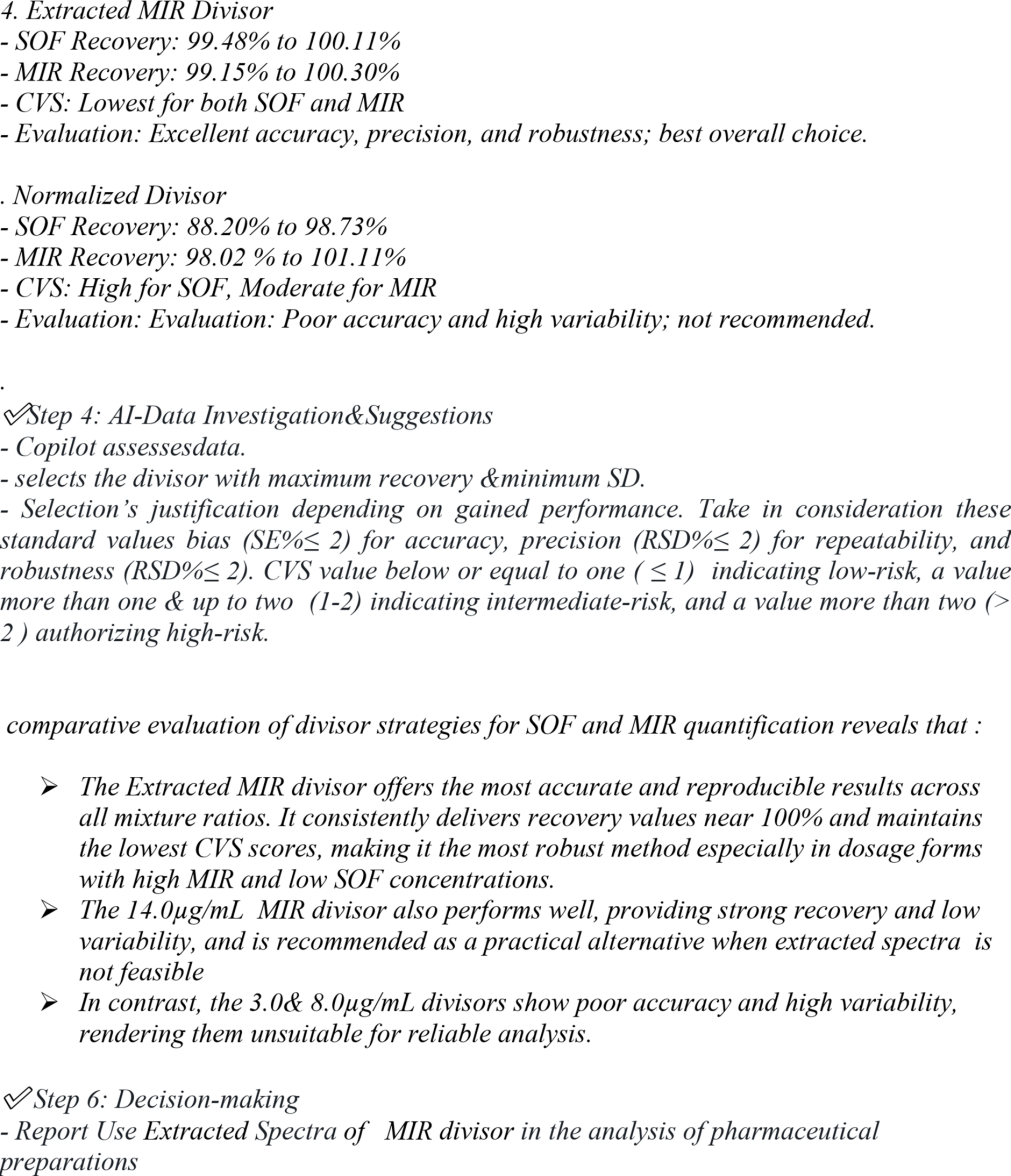
Steps for AI-Assisted Evaluation of Divisor Impact Using Microsoft-Copilot

For optimal spectral resolution and quantification of SOF and MIR, it is recommended to utilize the extracted MIR spectrum as a divisor, as this approach effectively eliminates interference and stabilizes the baseline and ultimately provides more reliable and precise quantification, ensuring robust results in complex sample matrices with minimum manipulation steps and no need for the step concerning calculation of the postulated amplitude.Additionally, when employing concentration-dependent divisors, selecting the highest concentration within the linearity range 14.0 µg/mL can enhance analytical accuracy by reducing spectral noise. Although this choice may slightly decrease sensitivity, it gives good and satisfactory results regarding accuracy and precision.

## Methods validation

Validation following ICH-guidelines was carried out^36^, with evaluation of critical parameters including sensitivity/linearity/accuracy/precision/limits of detection (LOD)/limits of quantification (LOQ)/robustness. It is worth noting that slight changes (± 0.1 nm), regarding scanning λ, was employed for robustness examination. A comprehensive summary of the validation results is provided in Table 2.Analysis’s results of different-ratios lab-prepared mixes, using normalized, concentration-dependent, and extracted MIR as divisors are presented in Table 1, & assured methods’ specificity. The most favorable outcomes were achieved when MIR at 14.0 µg/mL and extracted MIR were employed as divisors, as illustrated in Table 1.The proposed approach effectively analyzed the specified medicines in their respective tablet formulations. Table 3. Moreover, a statistical comparison was conducted between the results acquired from the studied methods and those from the official methods^37^.The calculated t and F values were observed to be lower than their respective theoretical values, indicating that there was negligible disparity between the studied and official methods in terms of accuracy and precision, as detailed in Table 4.

**Table 2:** Calibration regression parameters and method validation results for the proposed spectrophotometric analysis of SOF and MIR.

Parameter	SOF	MIR
Wavelength (nm)	D^1^ (222.0 nm)	D^0^ (249.0 nm)
**Calibration range (µg/mL)**	2.5–25.0	1.5–15.0
**Slope**	−0.031	0.0697
**SE of slope**	0.000331	0.000337
**Intercept**	−0.0031	0.0145
**SE of intercept**	0.004949	0.003068
**SD of residuals (S**_**y/x**_ **)**	0.00593	0.00353
**Correlation coefficient**	0.9997	0.9999
**LOD**	0.63	0.17
**LOQ**	1.91	0.51
**Accuracy**	100.06 ± 0.43	100.01 ± 0.53
**Repeatability (%RSD)** ^**a**^	0.39	0.65
**Inter-day precision(%RSD)** ^**b**^	1.36	0.87
**Robustness (%RSD)** ^**c**^	0.22	0.18

^a^ Relative standard deviation of three different concentrations repeated three times within the day of SOF (5.0,7.5 and 15.0 µg mL^− 1^) and MIR (9.0,12.0 and 15.0 µg mL^− 1^).

^b^Relative standard deviation of three different concentrations repeated three times in three successive days of SOF (5.0,7.5 and 15.0 µg mL^− 1^) and MIR (9.0,12.0 and 15.0 µg mL^− 1^).

^c^ Relative standard deviation of three determinations with change in scanning wavelength (± 0.1 nm).

**Table 3:** Results obtained using the optimal divisorMIR (14 µg/mL) for the determination of SOF and MIR in pharmaceutical dosage form.

Dosage Form	Drugs	Concentration (µg/mL)	Found conc*	Mean ± RSD ^a^
MEGATAS-S 25^®^ tablets, batch no. 1NP25CK02	MIR	14.00	14.03	100.23 ± 0.93
	SOF	2.80	2.84	101.32 ± 1.02

*Average of three determination.

**Table 4:** Statistical comparison between the results obtained by the proposed spectrophotometric method using the best scenario and official methods^37^ for the determination of SOF and MIR in pure powder form.

Items	SOF		MIR	
	D^1^ (222.0 nm)	Official method	MIR at 249.0 nm	Official method
**Mean**	99.59	99.35	99.95	100.00
**SD**	1.46	1.67	0.86	0.63
**RSD**	1.46	1.67	0.86	0.63
**n**	6	5	6	6
**Variance**	2.1316	2.7889	0.7369	0.3969
**Student’s-t test** ^**a**^ (1.812)	0.251		0.111	
**F-test** ^a^ (5.05)	1.308		1.863	

^a^ The corresponding theoretical values of t and F at *P* = 0.05.

## Sustainable Smart Analytical Chemistry (SSAC)

The concept of SSAC aims to bridge scientific innovation with global sustainability objectives, as shown in **Figure ****S1**. It builds upon GAC, WAC, and the emerging paradigm of smart analytical methods, which integrate AI into method design and optimization while connecting with SDG goals. In essence, SSAC integrates GAC, WAC, AI, and alignment with the SDGs to develop analytical methods that are concurrently efficient, innovative, and globally sustainable.

To estimate the degree of alignment of our established technique with GAC and WAC principles, as well as its level of innovation, we employed the MA Tool. This tool provides unified sustainability and performance evaluation without requiring multiple independent assessment systems. It comprises 51 structured questions with predefined answer choices: the first 21 assess greenness (adapted from the GEMAN tool)^32^; 10 examine applicability and practicality (based on the BAGI tool)^33^; 10 evaluate ruggedness and performance validity (from the RAPI tool)^34^; and the last 10 assess innovation (from the VIGI tool)^35^. The cumulative results are presented in three color-coded domains—green, blue, and red—each reflecting different sustainability dimensions. The overall Whiteness Score (A-score) is calculated as the average of these domains.

**Fig. 6:**
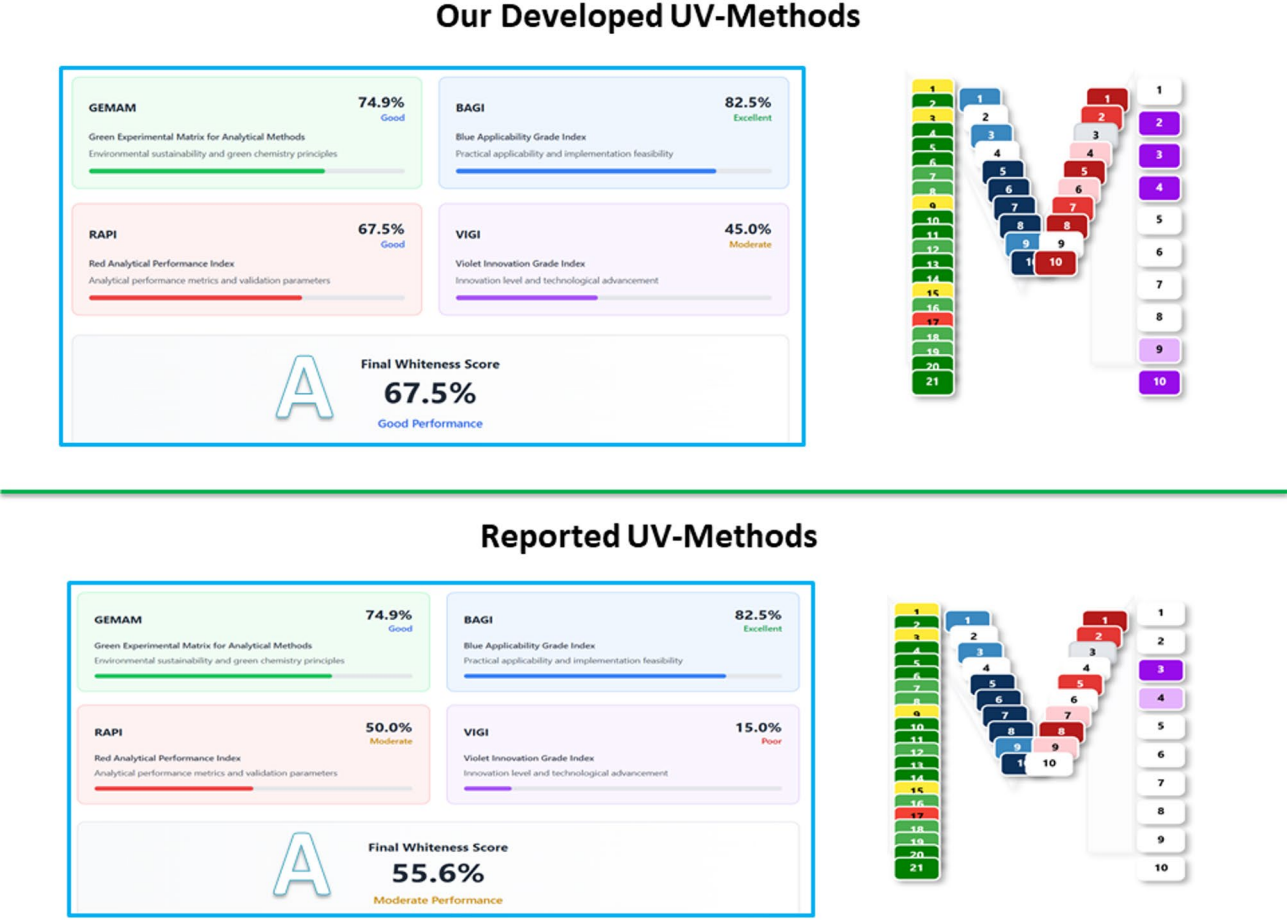
Comparative scores obtained from the MA Tool for the developed UV-spectrophotometric method and the reported method across GEMAN, BAGI, RAPI, VIGI domains, and the overall Whiteness Score (A-score).

Application of the MA Tool to both our newly developed UV-spectrophotometric method (optimized using AI) and previously reported UV method^12^ revealed distinct advantages of the proposed approach. We selected the reported UV method for comparison as it employs the same analytical technique, adhering to the guidelines for effective practice of sustainability metrics outlined in the systematic review (2020–2025)^38^, particularly rule that mandates fair and transparent comparisons on a like-for-like basis within the same technique. The GEMAN score was identical for both methods (74.9), as the solvent and sample preparation were the same. The BAGI score was also equivalent (82.5). However, differences emerged in other domains: the RAPI score was higher for our method (67.5 vs. 50.0), reflecting improved sensitivity, lower LOD values, and reduced bias during validation. The VIGI score strongly favored our method (45.0 vs. 15.0), emphasizing the innovative contribution of AI-assisted optimization. Consequently, the overall A-score was substantially higher for our method (67.5 vs. 55.6), as illustrated in Fig. 6. Detailed score in **Table ****S1****.**

Beyond methodological advances, both methods demonstrate a comparable degree of sustainability alignment, though the AI integration in our approach enhances innovation without altering the core environmental or practical profiles.

According to the SAMI framework, both our developed and the reported UV methods demonstrate a balanced sustainability profile, with an overall score of 35%, classifying them as sustainable. Both methods strongly fulfill several SDGs due to their low-cost, solvent-efficient design and practical applicability in pharmaceutical analysis, while remaining neutral in areas unrelated to their scope and violating only one due to gender representation in the research team. Specifically, they strongly fulfill SDG 3 (Good Health and Well-being) by providing an affordable, point-of-care compatible tool for drug monitoring in clinical settings, enhancing accessibility to therapeutic assessments for overactive bladder treatments. They also strongly fulfill SDG 8 (Decent Work and Economic Growth) with high practicality (> 75% in metrics like BAGI), offering cost-effective solutions that support industrial pharmaceutical quality control and economic efficiency. For SDG 12 (Responsible Consumption and Production), the methods achieve strong fulfillment through approximately 75% greenness (via GEMAN), minimizing solvent use and waste in line with green analytical chemistry principles. Similarly, SDG 13 (Climate Action) is strongly fulfilled, reflecting low energy demands.

The methods fulfill SDG 1 (No Poverty) by indirectly reducing expenses through efficient, instrument-minimal techniques suitable for resource-limited labs; SDG 4 (Quality Education) via their applicability in university research and training with advanced yet accessible instrumentation; SDG 10 (Reduced Inequalities) through lab-based methods that promote global quality standards and indirectly mitigate disparities in pharmaceutical access; SDG 11 (Sustainable Cities and Communities) with minimal waste, pollution, and noise; and SDG 17 (Partnerships for the Goals) due to collaboration between researchers from two countries (Egypt and Syria). They remain neutral for SDGs unrelated to their pharmaceutical focus, including SDG 2 (Zero Hunger), SDG 6 (Clean Water and Sanitation), SDG 7 (Affordable and Clean Energy), SDG 9 (Industry, Innovation, and Infrastructure), SDG 14 (Life Below Water), SDG 15 (Life on Land), and SDG 16 (Peace, Justice, and Strong Institutions). However, they violate SDG 5 (Gender Equality) due to unequal and underrepresented gender balance (< 25% of one gender) in the authorship and likely the research process, highlighting a trade-off where social inclusivity could be improved without compromising analytical performance. Globally, efforts to support women in research are essential to address such imbalances.

**Fig. 7:**
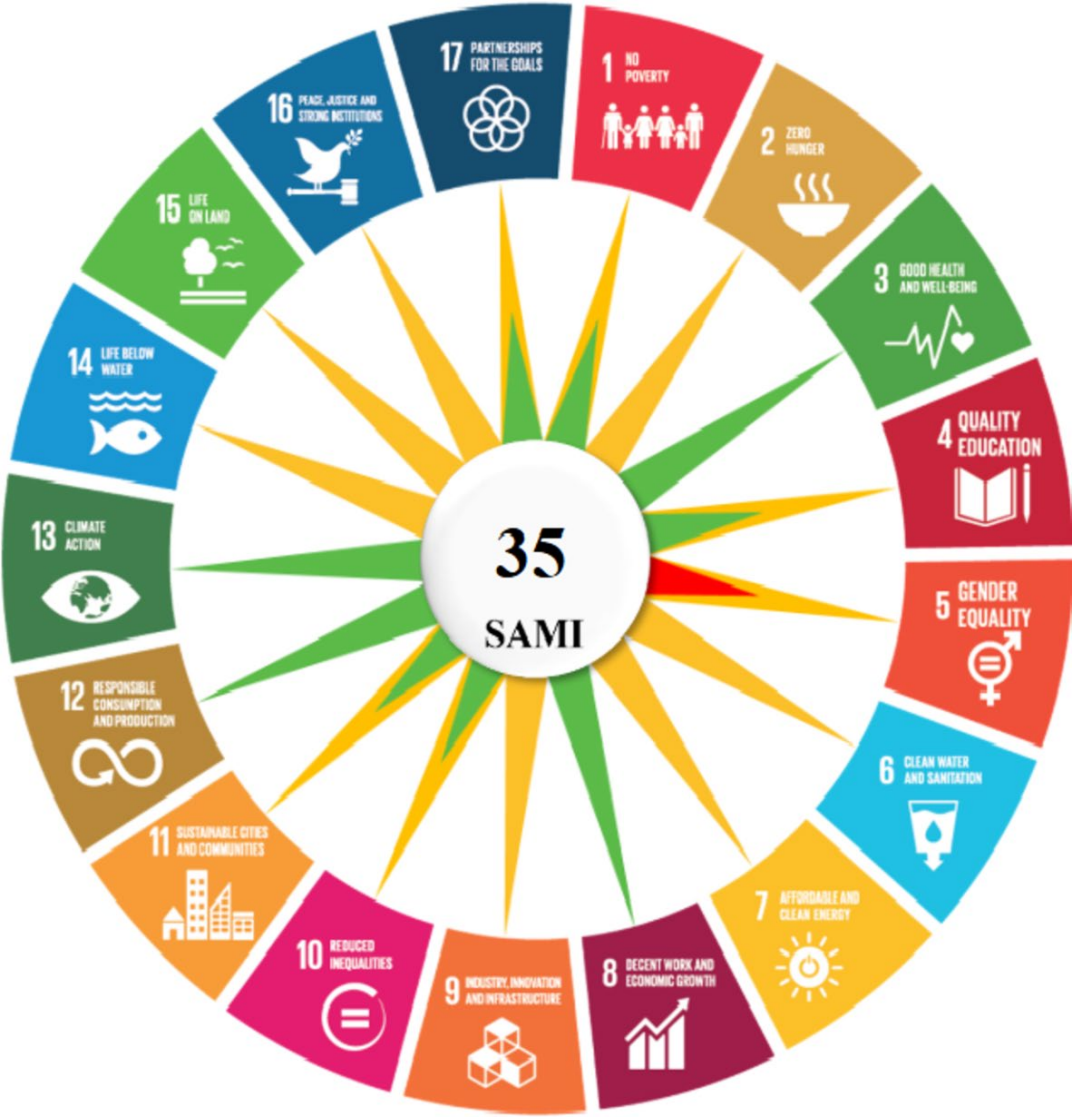
SAMI pictogram showing the shared sustainability profile for both developed and reported UV methods across 17 SDGs (green: fulfilled; yellow: neutral; red: violated), with a net score of 35 in the center.

These UV methods illustrate SAMI’s ability to capture the methods’ strengths in environmental and economic sustainability, while identifying opportunities for enhancement in social dimensions^39,40^. The AI integration and green focus make our approach a model for smart analytical chemistry. The SAMI visualization (**Figure. 7**) shows predominant green sectors for fulfilled SDGs, with limited red for the violation and yellow for neutrals, underscoring its positive net contribution to sustainable development. Detailed score in **Table S2.**

## Conclusion

In conclusion, the study highlights the critical role of divisor selection in optimizing spectrophotometric methods for pharmaceutical analysis. Among the evaluated approaches, the extracted spectra of MIR and highest MIR concentration as divisor 14.0 µg/mL yielded the best recoveries with minimal variability, indicating superior method performance. Using extracted MIR as a divisor has a privilege over 14.0 µg/mL since it has lower manipulation steps. The cumulative validation score (CVS) showing low risk of high variability and high analytical stability of these recommended divisors. The extraction of MIR’s zero-order spectra further enhanced accuracy and robustness, supporting the reliability of this approach. The procedure presented in this work does not need necessitate any expensive instrument and easy applicable on the built in software of the spectrophotometer and no other sophisticated programmes were used or purchased. The method could be functionalized in binary mixtures’ analysis in any quality-control laboratory. Integrating AI, green-chemistry principles, and white-analytical frameworks proved effective in developing sustainable and efficient analytical techniques. Overall, this methodology aligns with the objectives of Sustainable & Smart Analytical Chemistry, promoting both scientific excellence and ecological responsibility.

## Spectral resolution recommendation

For optimal spectral resolution and quantification of the binary mixtures:

- Use the Extracted zero order spectrum obtained via resolution method as a divisor to eliminate interference and stabilize the baseline, especially in asymmetric mixtures. It offers minimum manipulation steps.
- When applying concentration-dependent divisors, selecting the highest concentration within the linearity range is recommended, as it reduces spectral noise and improves accuracy though it may slightly reduce sensitivity.

## Future recommendation

Future recommendations for integrating AI into advanced spectrophotometric methods emphasize its potential to revolutionize analytical processes through enhanced efficiency and accuracy. To maximize these benefits, it is essential to supply AI systems with well-defined analytical standards, concentration limits, and validation thresholds. These parameters serve as critical guidelines that help prevent misinterpretation of data and ensure that analyses adhere to ICH guidelines and regulatory requirements and standards. AI tools excel in rapid pattern recognition, unbiased data evaluation, and consistent decision-making, making them invaluable assets in modern analytical workflows. As technology advances, continuous validation and calibration of AI models are necessary to maintain reliability and accuracy across diverse applications. Additionally, incorporating user training and transparent algorithms will foster greater confidence in AI-assisted analysis. Overall, adopting AI in spectrophotometry promises to streamline operations, reduce human error, and improve overall analytical quality in the future. For future studies, we aim to refine spectrophotometric methods for drug analysis in plasma, specifically addressing challenges posed by plasma protein interference and SOF peak overlap. Additionally, the application of standard addition enrichment will enable accurate quantification of the drug across its C_max_, enhancing the method’s reliability and clinical utility.

## Supplementary Information

Below is the link to the electronic supplementary material.

Supplementary Material 1

## Data Availability

All data generated or analyzed during this study are included in this published article.
