# Supplementary material for: Integrating Artificial intelligence within sustainable smart analytical chemistry for analyzing the divisor impact on UV-spectrophotometric efficiency of solifenacin and mirabegron combination
Source: Sci Rep. 2026 May 1;16:14022. doi: 10.1038/s41598-026-44688-x (PMC13134955; doi:10.1038/s41598-026-44688-x)
Supplement: Supplementary file 1 — Supplementary Material 1 [file 41598_2026_44688_MOESM1_ESM.docx]

**Supplementary Materials**

**“Integrating Artificial Intelligence within Sustainable Smart Analytical Chemistry for Analyzing the Divisor Impact on UV-Spectrophotometric Efficiency of Solifenacin and Mirabegron Combination”**

Hayam M. Lotfy^a^, Reem H. Obaydo^b^, Mahmoud A. Tantawy^c^, Aya A. Mouhamed^a*^

*^a^Pharmaceutical Analytical Chemistry Department, Faculty of Pharmacy, Cairo University, El-Kasr El-Aini Street, 11562 Cairo, Egypt*

^b^Department of Analytical and Food Chemistry, Faculty of Pharmacy, Ebla Private University, 22743, Idlib, Syria

^c^Department of Medicinal Chemistry and Pharmacognosy, College of Pharmacy, Qassim University, Buraydah 51452, Saudi Arabia

***Corresponding authors:**

Aya Ahmed Mouhamed: aya.ahmed@pharma.cu.edu.edg

**
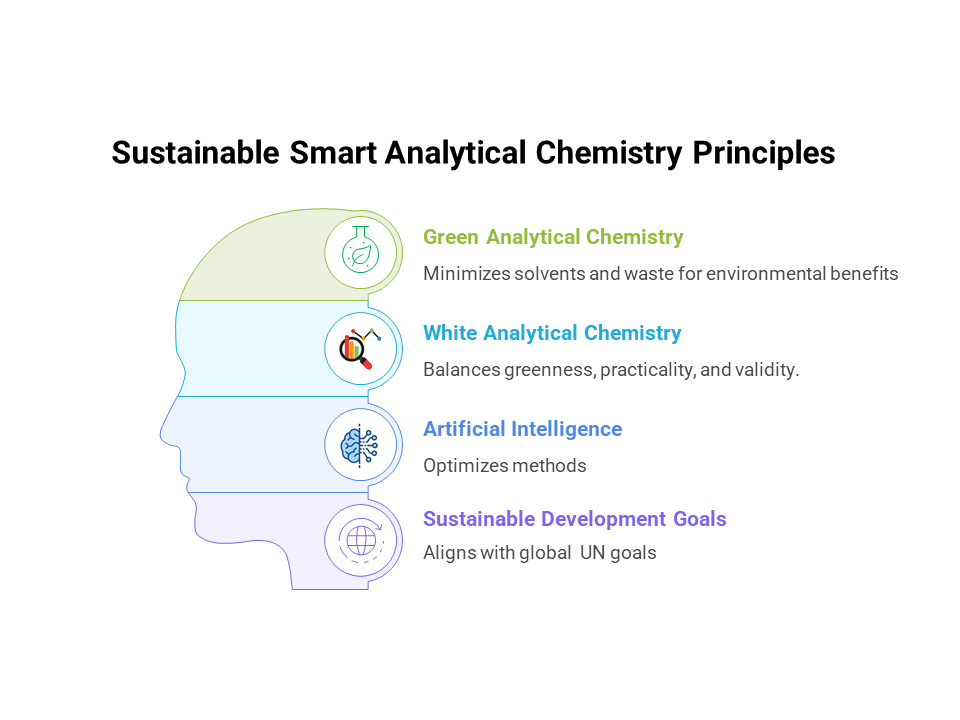
**

**Figure S1:** Sustainable Smart Analytical Chemistry Principles.

**Table S1.** MA Tool Scores for the Proposed and Reported UV Methods

| **Assessment Domain** | **Sub-Index Tool** | **AI-Optimized UV Method** | **Reported UV Method [9]** |
| --- | --- | --- | --- |
| Greenness | GEMAN | 74.9 | 74.9 |
| Practicality | BAGI | 82.5 | 82.5 |
| Analytical Performance | RAPI | 67.5 | 50.0 |
| Innovation | VIGI | 45.0 | 15.0 |
| **Overall Whiteness Score** | **A-Score** | **67.5** | **55.6** |

**Table S2.**  SAMI Results

| **Metric** | **AI-Optimized UV Method** | **Reported UV Method [9]** |
| --- | --- | --- |
| Net SAMI Score | 35% | 35% |
| Classification | Sustainable | Sustainable |
| Strongly Fulfilled SDGs | 3, 8, 12, 13 | 3, 8, 12, 13 |
| Moderately Fulfilled SDGs | 1, 4, 10, 11, 17 | 1, 4, 10, 11, 17 |
| Neutral SDGs | 2, 6, 7, 9, 14, 15, 16 | 2, 6, 7, 9, 14, 15, 16 |
| Violated SDG | 5 | 5 |
